# Structural barriers to maternity care in Cameroon: a qualitative study

**DOI:** 10.1186/s12978-024-01834-w

**Published:** 2024-07-19

**Authors:** Alfonsus Adrian Hadikusumo Harsono, Christyenne Lily Bond, Comfort Enah, Mary Glory Ngong, Rahel Mbah Kyeng, Eric Wallace, Janet M. Turan, Jeffery M. Szychowski, Waldemar A. Carlo, Lionel Neba Ambe, Gregory Halle-Ekane, Pius Tih Muffih, Alan Thevenet N. Tita, Henna Budhwani

**Affiliations:** 1 Department of Obstetrics and Gynecology, University of Alabama at Birmingham, Birmingham, AL USA; 2 Division of Gastrointestinal Surgery, Department of Surgery, University of Alabama at Birmingham, Alabama, USA; 3 Intervention Research and Implementation Science (IRIS) Lab, College of Nursing, Florida State University, Tallahassee, FL USA; 4 School of Nursing, College of Health Sciences, University of Massachusetts Lowell, Massachusetts, USA; 5 Cameroon Baptist Convention Heath Services, Bamenda, Northwest Region Cameroon; 6 Division of Nephrology, Department of Medicine, University of Alabama at Birmingham, Birmingham, AL USA; 7 School of Public Health, University of Alabama at Birmingham, Birmingham, AL USA; 8 Center for Women’s Reproductive Health, University of Alabama at Birmingham, Birmingham, AL USA; 9 Division of Neonatology, Department of Pediatrics, University of Alabama at Birmingham, Birmingham, AL USA; 10 Regional Delegation of Public Health, Bamenda, Northwest Region Cameroon; 11 University of Buea, Molyko, Buea, Cameroon

**Keywords:** Maternal health, Qualitative study, Cameroonian Women, Focus Group Discussions, Childbirth Care

## Abstract

**Background:**

The maternal mortality and perinatal mortality rate in Cameroon are among the highest worldwide. To improve these outcomes, we conducted a formative qualitative assessment to inform the adaptation of a mobile provider-to-provider intervention in Cameroon. We explored the complex interplay of structural barriers on maternity care in this low-resourced nation. The study aimed to identify structural barriers to maternal care during the early adaptation of the mobile Medical Information Service via Telephone (mMIST) program in Cameroon.

**Methods:**

We conducted in-depth interviews and focus groups with 56 key stakeholders including previously and currently pregnant women, primary healthcare providers, administrators, and representatives of the Ministry of Health, recruited by purposive sampling. Thematic coding and analysis via modified grounded theory approach were conducted using NVivo12 software.

**Results:**

Three main structural barriers emerged: (1) civil unrest (conflict between Ambazonian militant groups and the Cameroonian government in the Northwest), (2) limitations of the healthcare system, (3) inadequate physical infrastructure. Civil unrest impacted personal security, transportation safety, and disrupted medical transport system. Limitations of healthcare system involved critical shortages of skilled personnel and medical equipment, low commitment to evidence-based care, poor reputation, ineffective health system communication, incentives affecting care, and inadequate data collection. Inadequate physical infrastructure included frequent power outages and geographic distribution of healthcare facilities leading to logistical challenges.

**Conclusion:**

Dynamic inter-relations among structural level factors create barriers to maternity care in Cameroon. Implementation of policies and intervention programs addressing structural barriers are necessary to facilitate timely access and utilization of high-quality maternity care.

## Introduction

Reducing both maternal and newborn mortality is a high priority target in the Sustainable Development Goals (SDG) [1, 2]. The World Health Organization (WHO) aims for the reduction of global maternal mortality rate (MMR) to less than 70 per 100,000 live births by 2030 [3]. Insufficient care during labor and the first week after childbirth poses the highest danger to both mothers and newborns, leading to increased maternal and neonatal mortality due to complications, such as hemorrhage, hypertensive disorders and sepsis [1, 4–6]. Although prevention and management of such complications have been attempted and decreased the global MMR by 38% from 2000 to 2017 (342 to 211 deaths per 100,000 live births), the recent rate is still beyond the target [3, 5].

There is a significant disparity in maternal and neonatal mortality between high- (HIC) and low-income (LIC) countries, with outcomes in LICs being dramatically and consistently worse than their HIC counterparts [4, 7]. Approximately 99% of maternal mortality occurs in LICs, with the majority concentrated in Sub-Saharan Africa (SSA) [8]. Cameroon is a SSA country with an alarming MMR of 529 deaths per 100,000 live births, which is among the highest worldwide [2, 3, 9]. A comprehensive understanding of causes of these deaths is necessary to implement effective interventions, evidence-based health policy, and relevant clinical decision-making. Human and medical resources are less available in LICs as compared to HICs, and this resource gap contributes to deaths [4, 6, 10]. Other factors, such as socio-cultural and economic considerations, as well as low utilization of health services and slow health system adoption, are barriers to optimal maternal and neonatal care [1, 2, 11]. Among these barriers, structural barriers are critical because they impose challenges at a larger scale beyond the control of individuals and necessitate the allocation of government-level resources [12].

In Cameroon, lack of healthcare access, distance to health facilities, and shortage of healthcare staff and medical equipment are structural level barriers that have been identified [13–17]. Despite decades of attention from international agencies focused on reducing maternal deaths, outcomes are yet to significantly improve [18, 19]. Moreover, since 2016, civil unrest had worsened in the northwest and southwest regions of Cameroon due to the Anglophone crisis, which were spurred by the separatist group (Amba Boys) fighting for the independence of these regions, referred to as Ambazonia Republic [20, 21]. Civil disorder and disruption of the health system became the consequences [22]. Consequently, it is essential to revisit and document barriers to maternity care in the current context of Cameroon. This information will guide the development of new interventions to improve pregnancy outcomes [7].

As part of such interventions, we conducted a formative qualitative assessment to inform the adaptation of a mobile health (mHealth) support system and assess its acceptability in Cameroon while obtaining input from stakeholders by the local context and the lived experiences of Cameroonians [7, 23]. This mHealth system was based on a well-established working model that has been used in multiple states of the United States of America since 1969 including the University of Alabama Medical Information Service via Telephone (MIST) [7, 24]. We intended to adapt this intervention for mobile phones to reduce costs and increase network coverage using the existing infrastructure in Cameroon [15, 25]. The aim of this study was to identify current structural barriers to maternal care during the early adaptation of the mMIST program through in-depth interviews and focus groups with key stakeholders in Cameroon. Findings on individual and interpersonal barriers are presented elsewhere (under review).

## Materials and methods

This qualitative examination was the initial step in adapting the MIST line to the Cameroonian mMIST, a 24/7 mHealth support system for primary providers of pregnant women in Cameroon built upon stakeholders’ feedback and existing UAB schematic [7]. In the full protocol, our qualitative findings will be used in adapting MIST to mMIST, and thereafter the mMIST intervention will be tested in a stepped wedge cluster randomized controlled trial of sixteen health districts in the northwest region of Cameroon. Details about our full study are detailed in our published protocol manuscript [7]. Two methods of data collection were used, face-to-face in-depth interviews and focus group discussions (FGDs) using semi-structured guides with a total of 56 stakeholders [26]. Purposive sampling [26] was leveraged to include the various kinds of key stakeholders to represent real-life experiences of maternal care in Cameroon. We interviewed 12 maternity care providers, 6 Ministry of Health officials, 10 health system administrators and clinical staff, as well as 10 previously pregnant women with adverse outcomes to assess the local context and barriers to maternity care. Three FGDs with 18 currently pregnant women were also conducted, with 5–7 women in each group. Some recruited participants, including currently pregnant women, cancelled last minute and missed the interview due to unforeseen circumstances. Hence, enrollment of participants was conducted until data saturation was reached.

Data were collected between April and June 2021 in the northwest region of Cameroon. Interviews and FGDs were guided by five trained Cameroonian female research team members from the Cameroon Baptist Convention Health Services (CBCHS) and conducted using semi-structured interview guides, in English and Pidgin English. Facilitators were trained by experts in qualitative methods. Repeat interviews were not performed; therefore, no formal field notes were captured, nor participants were provided transcripts for comment. All interviews and FGDs were audio recorded, uploaded into our secure database, professionally transcribed, translated using cultural translation techniques, and then coded by two independent coders in Cameroon (MGN) and in the United States (CLB). One transcript per participant category was collaboratively coded (double-coded), with the codebook being modified as necessary to capture all pertinent themes. The remaining transcripts were individually coded, evaluated, and revised if discrepancies between coding were discovered. No feedback was provided by the participants regarding the findings.

Based on a modified Grounded Theory approach [27, 28], coding and analysis use a thematic analysis approach, in which a priori themes and sub-themes from theory and literature are supplemented with emerging themes “grounded” in the data. Coding was conducted using NVivo 12 software (Burlington, MA). A two-step coding scheme was used, a preliminary coding framework was developed based on topics from the interview guide, followed by second-level fine coding. Initially, transcripts were double-coded while adjusting the codebook in order to capture all relevant themes. After the codebook was finalized, the remaining transcripts were individually coded and carefully reviewed to reconcile any discrepancies. A total of 45 codes within 4 domains were revealed and further classified into sub-themes. We applied the Consolidated Criteria for Reporting Qualitative Research Studies (COREQ) checklist to present study results [29].

This study was evaluated and approved by the Cameroon Baptist Convention Health Services (CBCHS) Institutional Review Board (IRB, IRB2020-49) and the University of Alabama at Birmingham (UAB) Institutional Review Board (IRB-300006254). All participants provided written informed consent, and this study was conducted in compliance with the declaration of Helsinki.

## Results

### Descriptive statistics of study participants

The sample (Table 1) was largely female (75%), with the majority being previously or currently pregnant women (50%) with mean age of 35 ± 10 years. All of them self-identified as Cameroonian. Most of the participants were married (84%), and the majority (84%) had completed at least high school. There were slightly more participants who resided in rural (55%) compared to the urban settings. The average duration of interviews was 31 ± 13 min and that of FGDs was 41 ± 9.6 min.

**Table 1:** The demographic characteristics of the samples

Categories	Total	Maternity Providers	Previously & Currently Pregnant Women	Administrators	Ministry of Health
	**(** ***N*** ** = 56)**	**(** ***n*** ** = 12)**	**(** ***n*** ** = 10)**	**(** ***n*** ** = 10)**	**(** ***n*** ** = 6)**
**Highest level of education completed**					
Some primary school	9 (16.1%)	0	9 (32.1%)	0	0
Some high school	19 (33.9%)	7 (58.3%)	11 (39.3%)	1 (10%)	0
Technical schooling	1 (1.8%)	0	1 (3.6%)	0	0
Some university	2 (3.6%)	1 (8.3%)	0	0	1 (16.7%)
BS/BA or similar	11 (19.6%)	3 (25%)	5 (17.9%)	3 (30%)	0
Post graduate studies	13 (23.2%)	1 (8.3%)	1 (3.6%)	6 (60%)	5 (83.3%)
**Sex assigned at birth**					
Male	14 (25%)	3 (25%)	28 (100%)	8 (80%)	3 (50%)
Female	42 (75%)	9 (75%)	0	2 (20%)	3 (50%)
**Marital status**					
Married	47 (83.9%)	8 (66.7%)	23 (82.1%)	10 (100%)	6 (100%)
Not Married	7 (12.5%)	4 (33.3%)	3 (10.7%)	0	0
**Residence location**					
Village	31 (55.4%)	6 (50%)	20 (71.4%)	5 (50%)	0
City	25 (44.6%)	6 (50%)	8 (28.6%)	5 (50%)	6 (100%)
**Age** ^a^	35.1 ± 9.9	36.8 ± 10.2	29.4 ± 5.2	45.8 ± 10.8	44.5 ± 6.3

Data are all presented in n (%)

^a^Age is presented in Mean ± SD

### Structural themes

Identified structural barriers to maternity care in Cameroon from our research participants’ accounts converged on three common themes: 1) civil unrest, 2) health system, and 3) physical infrastructure. Each theme is further explored in the following section and illustrated with quotes. Additional illustrative quotes for the themes and sub-themes can be found in Table 2.

**Table 2:** Additional illustrative quotes for structural themes and sub-themes

Theme	Sub-theme	Quote
1. Persistent Civil Unrest	1A. Threats to Personal Security	*The common complications especially where I work is previous CS (Caesarean section) and then since such cases cannot be handled in a place where there is no theatre, so the only option is to go to the district hospital… one of the things they fear so much is that the district hospital is also close to the military camp…* -Maternity provider, 44, Female
	1B. Unsafe Transportation	*…and also, one of the most important things that needs to be done is to bring the current crisis in the region to an end …because there are some women who have delivered in the house not because they did not have money… but there was no way to get to the hospital* -Administrator, 30, Male
	1C. Disruption of Medical Transport System	*It becomes very difficult… when they have premature labor… and you have to transport it now to the hospital and the road is not accessible…, at times you take the baby and then they resend back maybe because of the nature of the crisis* -Maternity provider, 36, Female
2. Inadequate Health System	2A. Lack of Maternity Care Providers	*It is very strenuous and difficult because working there as a mid-wife alone, you will do everything alone because you do not have somebody that you will provide task… you do everything and if there is a labor you need to work from start to delivery* -Maternity provider, 36, Female
	2B. Lack of Specialists	*I was with a midwife… they can only manage to give me antibiotics until the day the doctor will come and do the work or the surgery* -Previously pregnant woman, 32, Female
	2C. Lack of Commitment to Evidence-based Practices	*We could also want to encourage regular refresher courses for maternal health care providers… because at times you may have the equipment you may have the maternal (providers) but without (committed) personnel you will not be able to do it effectively* -Maternity provider, 28, Male
	2D. Critical Shortage of Medical Equipment	*…when the children came out all was alive, but I see that they did not have enough machine (incubator)… to sustain my children, so they passed away… if (only) they had machines… my children might still be alive today* -Previously pregnant woman, 29, Female
	2E. Poor Communication System between Health Units	*I had this woman who was referred from a health center due to severe preeclampsia… she came in unstable… and we were unable to auscultate the fetal heart rate. …out of sudden the woman just arrested, We struggle pushing to theatre while doing CPR …all efforts proved abortive, so we ended up with a maternal death… if they had called me 2 days earlier, maybe it would have been different* -Maternity provider, 49, Female
	2F. Lack of Incentives to Provide Quality Care	*…because there is little budget for health care… the UNO (United Nations Organization) expect that each country should budget at least 1% of their GDP for health but I think in Cameroon, we do not even budget up to 0.5. The budget is usually so small that hardly is enough…* -Administrator, 49, Male
	2G. Inadequate Data Collection Systems / Processes	*…documentation is poor because all documentation is manual, and it takes time… you can imagine, if a patient comes in to register for antenatal care when they are going to [register anew]… when they register, they are going to the laboratory you register anew, they are going to pay, you register anew [again] …everything is done anew so that takes a lot of staff time. Whereas if we had an electronic system where once a client is registered, the name is already in the system and you need to add any information, you just add* -Administrator 410, age 49, Male
3. Insufficient Physical Infrastructure	3A. Inadequate Facilities Lead to Logistical Challenges	*…usually in very remote communities… people will prepare something in form of a ladder …a local stretcher and carry the woman on. …growing up, I witnessed a situation wherein we had this wheelbarrow. They pushed women in the wheelbarrow to the health center* *-Administrator, 30, Male*
	3B. Power Outages	*We have epileptic power supply, unstable network and if we don’t have electricity the phones will not be charged* -Maternity provider, 28, Male

### Theme 1: persistent civil unrest

Civil unrest, which consists of the Anglophone regional crisis, has been a persistent barrier to maternity care for years in Cameroon. Pregnant women in focus group discussions explained that unpredictable and periodic violent confrontations between the separatists and the regular military threatened civilians’ personal security, made transportation unsafe, and disrupted medical transport systems.

#### Sub-theme 1A: threats to personal security

Focus group discussions with pregnant women revealed that tensions arising from the civil unrest frequently force civilians to flee for safety. Many people may not have the security in their own homes, let alone access to healthcare facilities for maternity care.

*…on several occasions they (the military) have broken my door, entered my house, and scattered everything but it is always fortunate that when they are coming, we are aware in the quarter so, I pack a few of my belongings; carry my little children coupled with my pregnancy and run to the bush…*

-Focus group, currently pregnant woman

A currently pregnant woman described how such tensions led to potentially fatal complications in her previous pregnancy.

*When I was 3 months pregnant for my baby, military attacked my quarter and in the course of running, I had slight bleeding… We were in the market on Saturday, and they started shooting, we left and ran home. Then from Monday, the baby started coming out when it was just 34 weeks.*

*-*Focus group, currently pregnant woman

#### Sub-theme 1B: unsafe transportation

Focus group participants indicated that violent confrontations could occur anywhere at anytime, and with gunshots fired in public places, transportation is especially difficult.

*…the military saw them* (Amba boys) *and started firing guns... Now for us, there was no way to run… the boys instead, they ran and fell on top us with their guns on their back… there was no bike, every place was just like a dead zone. I rushed to one woman… and we had to trek from Mveh under the heavy rain to this hospital.*

-Focus group, currently pregnant woman

According to focus group participants, some areas where the separatist camps are located, were off-limits to civilians. Therefore, travel time to the hospital had significantly increased, and some women were forced to deliver in bushes which put the mothers at risk for complications and in fatalities.

*This lady was referred from Ndu and took almost 2 weeks to reach (the hospital)… there were gunshots, and they get back to the bush. The man had carried her on the back and was tired… and finally when they reached the hospital the baby was no more. The woman was tired with swollen legs, and finally she died… these are just 2 I have seen, what about those who have died that I have not seen?*

-Maternity provider, 49, Female

#### Sub-theme 1C: disruption of the emergency medical transport system

To further complicate the situation, the crisis has halted previously operating medical transport services.

*…before the northwest and southwest crisis erupted, the CBCHS used to have helicopters or planes to transport women up who were in distress from remote areas to the hospitals ….but with the coming of the crisis the planes are no longer being flown.*

-Administrator, 64, Male

*When things were normal… we will immediately try to rescue with the helicopter that we had at that time… the crisis came and stopped even the vehicles we could use to go rescue the women; the roads were blocked, no way to go do that…*

-Administrator, 44, Male

### Theme 2: inadequate healthcare system

Challenges identified within the Cameroonian health system included critical shortages of skilled personnel and medical equipment, lack of commitment to evidence-based practices, power outages, poor provider or institutional reputation related to clients’ previous negative experiences, flawed communication systems between health units, low healthcare worker compensation affecting quality of care, and subpar documentation system for feedback.

#### Sub-theme 2A: lack of maternity care providers

Hospitals and health facilities are currently experiencing serious staffing shortages which threaten access to maternity care in the communities they serve. Finding a midwife or nurse in some parts of Cameroon might be difficult, and if they were available, there might only be one single person managing the entire health facility.

*Staffing in health centers is not the best, so it’s in rare situations where you may have two people available… In the maternity (unit) most of the times it’s one person.*

-Administrator, 37, Female

Participants said even when health personnel were available, they might not be trained to provide maternity care, much less identify and manage common maternal and fetal complications.

*…sometimes, a woman has seen a provider 4 times with an elevated blood pressure, and they have not done a complete urinalysis to find out whether that is impacting the organs of the pregnant woman… they end up having eclampsia or pre-eclampsia and HELLP syndrome.*

-Administrator, 64, Male

#### Sub-theme 2B: lack of specialists

Our participants noted a lack of sufficient specialists, such as obstetricians and pediatricians, as well as nurses specialized in neonatal care, to keep up with the increasing number of deliveries and to cover all of the healthcare facilities.

*I think service providers, nurses, midwives, and our clinicians need to have skills on newborn care. There seem to be increase in deliveries with children who are underweight, and they have to be in the incubator... there is a need to improve on the skills of our service providers on how to manage these neonates so that they gain the required weight and thrive*.

-Administrator, 47, Male

*…we also need specialists, people who will take care of complications when they occur… I think CBCHS has gynecologists only in 3 facilities whereas we have over 8 hospitals… We do not have pediatricians who will take care of newborns if they have certain problems.*

Administrator, 49, Male

#### Sub-theme 2C: lack of commitment to evidence-based practices

Providers acknowledge that some clinicians still perform outdated practices, emphasizing the need for standardized trainings such as basic (BLSO) and advanced life support in obstetrics (ALSO) aside from annual refresher courses for continuous professional development. Some providers do not want to update their skills with current scientific evidence, resulting in life-threatening consequences for patient care.

*…a patient had an IUFD and the baby was big… they did not want to go ahead and operate… they induced her and… when she delivered the head she had a stuck shoulder and it was not easy… we did have knowledge on how to deliver this four kilogram baby*

-Maternity provider, 47, Female

*…(during) my first pregnancy, … in the process of delivery in the hospital, (I) labored for 3 days. After that, (I was) induced labor on the third day and finally they had to take me for operation which I even lost the baby… the carelessness was really too much… they want to remain at the level at which they are….*

-Focus group, currently pregnant woman

#### Sub-theme 2D: critical shortage of medical equipment

Some hospitals lacked the appropriate medical equipment to provide maternity and newborn care altogether, while others had substandard or even defective equipment. Consequently, patients had to be referred to other hospitals, delaying treatments.

*I also had an experience where I gave birth and got home, the baby made 2 weeks; I came to a small hospital. It showed that they should put the baby on oxygen whereas they did not have the oxygen… and the child finally died.*

-Focus group, currently pregnant woman

Healthcare providers noted that the lack of a central blood bank system in the region contributed to the challenges in the management of postpartum hemorrhage.

*…we have a very big challenge when it comes to managing post-partum hemorrhage because we don’t have a blood bank in the region... Most blood banks are found in the health facilities. …but they will not have a central blood bank that mobilizes blood from the population and distribute to all these other health facilities.*

-Ministry of Health, 33, Male

#### Sub-theme 2E: poor communication system between health units

Health care providers explained that each health unit in Cameroon has a designated level at which it can function, according to classification by the Ministry of Health. When they face limitations, they refer patients to the district, then the regional level, and the referral hospitals. However, communication across the various levels was flawed, resulting in treatment delays.

…*if we could have the means before you refer a case you already call and notify the hospital that you are sending to… so that they can prepare when the client comes. It is going to help reduce client waiting time and also help in reducing the poor outcomes we usually have.*

-Maternity provider, 28, Male

*It is only when that woman started bleeding profusely, that he tried to control the bleeding; he was unable to and rushed this woman to the health facility. …we had no idea that a case like that was even coming… These are things that if they would have called, you would have said ok, check the blood pressure… this is what you have to do to contract the uterus to prevent further bleeding… give magnesium sulphate and bring the woman directly to the hospital. We will start preparing blood, knowing that the woman is bleeding before the woman arrives the health facility. So, communication is essential.*

*-*Ministry of Health, 33, Male

#### Sub-theme 2F: lack of incentives to provide quality care

Policies that promote safe maternity practices must be reinforced at all levels from the top down. However, different health facilities are managed and staffed by different personnel who might have varied objectives. For reasons of prestige and financial advantage, some providers might wish to retain their patients in their care, even if they were incapable of managing them.

*There are some providers who think they know it all especially some of the traditional birth attendants, even when they have difficult cases, it is difficult for them to refer. They want (to) swell their statistics and with the (up)coming of the performance-based financing many of them want to keep their patients.*

-Administrator, 39, Male

*… As I said, one of the challenges we have at the level of the periphery is the lack of motivation. Some of them are not paid, they go for months without being paid. Some of them depend on other income generating activities. So, setting up this program, they need to find their heads up, how is it going to help them to boost their activities at the periphery.*

-Ministry of Health, 45, Female

#### Sub-theme 2G: inadequate data collection systems / processes

Some participants argued effective documentation is burdensome because it is an additional task for providers who are already overburdened. Nonetheless, such information is fundamental as feedback on current practices and to continuously inform and enhance maternal and neonatal health.

*…quality data, which can be analyzed will give us the information that we need… Staff frequently are not collecting the data we need because we collect data manually. …when it looks cumbersome and somebody needs to choose between putting in the right data or taking care of a patient, they may choose to take care of the patient and the data is lost.*

-Administrator, 49, Male

*The data quality is not good, people are not trusted. As a typical example in 2016, there were very few deaths, …in a year maybe 13 or 14 deaths. …I said no, the community is showing that women are dying and being buried but it is not reported anywhere, it is only when they started tracking and finding out that there were so many unreported deaths in the community…*

-Ministry of Health, 45, Female

### Theme 3: insufficient physical infrastructure

Sufficient physical infrastructure has been a challenging factor in ensuring quality maternity care. This theme consists of inadequate facilities which lead to logistical challenges and unstable electricity issue.

#### Sub-theme 3A: inadequate facilities lead to logistical challenges

According to providers, gaps in the geographical distribution of health facilities exist, notably in rural communities where women are required travel long distances in an unfavorable condition to seek maternity care. This can be a substantial burden which also endangers both the mother and child.

*There are some women who will trek for more than 5 kilometers before they access a health center… it actually makes them feel reluctant to go… Some start antenatal care very late. Some will want to go two times or once before delivery. They will go at 36 weeks and at times there are complications that cannot even be rectified at that moment.*

-Maternity provider, 37, Female

The roads in remote areas are either non-existent or sometimes can only accommodate bicycles, forcing individuals to walk long distances through rugged terrains. Even in the city, the roads are very narrow and congested.

*…most do not even have access to do bikes or motorbikes and you can imagine the remote community that people can only trek long distances… and under very harsh conditions.*

-Administrator, 47, Male

#### Sub-theme 3B: power outages

The unstable electricity supply in Cameroon with frequent power outages adversely affects the operation of health facilities and may impair the quality of maternity services provided due to the inability to operate certain medical equipment (Table 2). Health care providers noted that in certain villages, power outages might last for days or even months which greatly diminish the capacity to accommodate new patients.

…the power shortage in Cameroon is a big problem… there is always failure in electricity. …to implement this project and function 24/7 as you rightly said, we should have in mind that we may look to alternative means…

-Maternity provider, 29, Female

It is possible that there will be (a situation) that… if electricity is off for some days or some months as is the case in our village now, then if you call when the phone is down, then you will not get to the person.

-Maternity provider, 44, female

## Discussion

Our study uncovered three main themes around structural or system level barriers affecting optimal maternity care in Cameroon: persistent civil unrest, inadequate healthcare system, and insufficient physical infrastructure. Our novel contribution is providing a contemporary account of the situation in the northwest region of Cameroon, an understudied setting that is currently in conflict, resulting in unique barriers. This qualitative work has potential to not only inform behavioral science but also clinical care under sociopolitical instability. Interviews with key stakeholders suggested that the regional civil unrest has had an enormous impact on access to maternity care. Previous research in Africa and the Middle East has underlined how political conflicts elevated MMRs in countries where they occur, aside from the spillover regional effects [30–38]. In-depth interviews provided specific examples of how the crisis in Cameroon negatively affects maternity care. Currently and previously pregnant women emphasized their inability to prioritize maternity care, because security of their own homes and fulfillment of their basic needs were unmet. Accessing health facilities was further hampered by the armed conflict, insecurity, and kidnappings. Study participants explained that since 2016, the civil unrest in the northwest region of Cameroon has posed as a significant barrier to maternity care [22]. Personal threats, unsafe transportation condition, and medical transportation insecurities were the specific causes. Our proposed telehealth mMIST system was intended to overcome this barrier by facilitating a more seamless maternity care referral system amid the political issue and insecurity.

Limitations of the healthcare system was identified as the second main structural barrier. Respondents highlighted the severe shortage of healthcare personnel in terms of both quantity and quality to provide quality maternity care. This resulted in unfavorable experiences for some pregnant women and a negative reputation for maternity care, which discouraged women from seeking care. Necessary testing to prevent complications, such as urinalysis for preeclampsia, were not carried out, indicating that further training and refresher courses are essential to maintain the quality of the healthcare service. From the patients’ perspectives, poverty and financial barriers majorly contributed to the low participation of healthcare seeking behavior during pregnancy [31, 32]. Governmental and non-governmental financial support in terms of reducing healthcare cost through subsidy and health insurance would be beneficial to increase healthcare access to those facing financial barriers [32, 33]. Notably, from healthcare providers’ perspectives, a common recommendation from all groups of stakeholders was for healthcare staff to upgrade their knowledge of current evidence-based practices and procedures including neonatal resuscitation and attend ongoing refresher courses. With the advent of performance-based financing, some respondents expressed concern that providers’ self-interest for financial advantage and even prestige may generate a conflict of interest and discourage patient-centered treatment. This finding conflicts with the growing body of literature on the effectiveness of performance-based financing strategy in reducing maternal and neonatal mortality [34].

In HICs, there is a pivot from primary care to specialized care resulting in a shortage of first-line providers and increased numbers of specialists. In LICs, particularly in Cameroon, there is a shortage of both. Hospitals and health facilities are currently experiencing serious staffing shortages which threaten access to maternity care and treatment in the communities they serve [35]. While primary providers are necessary to deliver first line care, specialists are necessary to address life-threatening complications. The shortage of obstetricians as well as pediatricians underscores the extreme lack of healthcare personnel. Our findings are consistent with those of previous research in the same region, which recognized shortages of skilled health personnel and deficiencies in education, training, and continuing education as barriers to reproductive healthcare awareness [13, 18]. Shortage of medical supplies, including blood products and unsteady power supply, also limited the provision of quality maternity care. Our mMIST intervention will address identified structural barriers through capacity building to bolster the knowledge and skills of first-line responders, staff, and providers within a well-integrated referral network. While, mMIST cannot eliminate the risk of losing healthcare professionals, the intervention will increase the capacity of health care providers. Additionally, by creating this interconnected network of providers, sharing of life-saving supplies between institutions can occur, which does not eliminate shortages but does increase the efficiency of use.

The literature also supports a key finding from our study underlining the need for effective communication, both within and between health facilities to assure timely acknowledgement of complications and avoid delays in appropriate care [36]. However, without a standardized referral protocol and a well-established transfer system, a systemic culture of downstream blaming and mistrust may flourish. Hence, developing an ecosystem where accountability is shared throughout the health system is imperative including debriefings when adverse events occur [37]. In addition to offering practical evidence-based information at the point-of-care, our proposed mMIST telehealth intervention aims to ease the referral process and facilitate inter-facility communication, including debriefings [7].

The final main structural barrier revolves around the issue of insufficiency of physical infrastructure in the country. According to our findings, due to the limited number of healthcare facilities, geographical distance to the nearest maternity care facility can be a substantial problem especially in rural areas. This reduces maternity care utilization and has been shown to significantly increase maternal and newborn mortality in prior studies [38, 39]. Further findings suggest that insufficient roads and transportation systems limit access to maternity care. Infrastructural development programs could be beneficial in addressing these issues. Alongside financial barriers and civil unrest, geographical barriers including physical infrastructures have contributed to the delay of accessibility. As discussed previously, mMIST could also address this accessibility problem by streamlining the referral system by providing a network of experts and first-line providers to provide guidance for management maternal and neonatal complications at local health facilities.

Limitations of our study should be considered. Qualitative data are not generalizable and reflect the subjective perceptions of the participants we were able to interview. Thus, the scope of structural problems captured herein may not reflect all structural barriers that exist in the region. Further studies on maternal health using quantitative or mixed method [13, 40] approaches that evaluate countrywide epidemiological data alongside feedback from pregnant women are recommended to complement our findings in order to establish a broader understanding of the barriers and decrease MMRs specifically in the Northwest region of Cameroon.

## Conclusion

In summary, structural barriers to maternity care in Cameroon as LIC involved the conflict, health system, and infrastructures. Although some barriers have been identified, newly discovered and more detailed barriers were identified in this study through first-hand experiences of key stakeholders. There is an urgent need to implement interventions to mitigate the high MMRs and improve the quality of maternity care. Our adaptation of the mMIST system is intended to address the aforementioned barriers.

## Data Availability

The datasets during and/or analyzed during the current study available from the corresponding author on request.
